# Moderated Online Social Therapy: A Model for Reducing Stress in Carers of Young People Diagnosed with Mental Health Disorders

**DOI:** 10.3389/fpsyg.2017.00485

**Published:** 2017-04-03

**Authors:** John Gleeson, Reeva Lederman, Peter Koval, Greg Wadley, Sarah Bendall, Sue Cotton, Helen Herrman, Kingsley Crisp, Mario Alvarez-Jimenez

**Affiliations:** ^1^School of Psychology, Australian Catholic UniversityFitzroy, VIC, Australia; ^2^Department of Computing and Information Systems, The University of MelbourneParkville, VIC, Australia; ^3^Orygen, The National Centre of Excellence in Youth Mental HealthParkville, VIC, Australia; ^4^Centre for Youth Mental Health, The University of MelbourneParkville, VIC, Australia; ^5^Orygen Youth HealthParkville, VIC, Australia

**Keywords:** youth mental health, early psychosis, psychosocial treatment, family intervention, online treatment, psychoeducation, online social networking

## Abstract

Family members caring for a young person diagnosed with the onset of mental health problems face heightened stress, depression, and social isolation. Despite evidence for the effectiveness of family based interventions, sustaining access to specialist family interventions is a major challenge. The availability of the Internet provides possibilities to expand and sustain access to evidence-based psychoeducation and personal support for family members. In this paper we describe the therapeutic model and the components of our purpose-built moderated online social therapy (MOST) program for families. We outline the background to its development, beginning with our face-to-face EPISODE II family intervention, which informed our selection of therapeutic content, and the integration of recent developments in positive psychology. Our online interventions for carers integrate online therapy, online social networking, peer and expert support, and online social problem solving which has been designed to reduce stress in carers. The initial version of our application entitled Meridian was shown to be safe, acceptable, and feasible in a feasibility study of carers of youth diagnosed with depression and anxiety. There was a significant reduction in self-reported levels of stress in caregivers and change in stress was significantly correlated with use of the system. We have subsequently launched a cluster RCT for caregivers with a relative diagnosed with first-episode psychosis. Our intervention has the potential to improve access to effective specialist support for families facing the onset of serious mental health problems in their young relative.

## Introduction

Severe mental health disorders most often have their initial onset during late adolescence and early adulthood which has several critical implications for the lives of family members (de Girolamo et al., 2012). In roughly 90% of cases, families provide emotional and instrumental support to the young person at the point of onset at a stage of development when most parents hold the expectation that the young person in their care is approaching the threshold of greater independence (Onwumere et al., 2011). As a consequence, when symptoms of a mental health disorder develop in their young relative, many family members are confused and overwhelmed regarding the nature and causes of changes in the young person's behavior, let alone the required interventions (Gleeson et al., 1999). Even when appropriate mental health care is accessed in a timely manner the process of diagnosis and initiation of treatment is often a source of considerable distress for family members (Jansen et al., 2015). All too often entry into mental health care is delayed. Delay is likely to worsen the prognosis and potentially prolong the uncertainty and helplessness for family members (Marshall et al., 2005).

If acute phase care is required, family members are then exposed to the reality of the diagnosis for the first time with significant associated anxiety and uncertainty regarding treatment options and the prospects for recovery. Emotional responses for carers are varied but there are some common themes including guilt, heightened anxiety, and, where recovery is prolonged, a sense of loss (Patterson et al., 2005; Onwumere et al., 2011). The day-to-day behavioral consequences of disorders such as mood and psychotic disorders and their treatments and their treatment are known to be associated with high rates of stress, distress, and depression in carers (Sobieraj et al., 1998; Addington et al., 2003). In short, family members are faced with their own significant mental health challenges. Less is known about the physical health impacts on family members, but research from carer research in dementia suggests that increased risks may arise (Damjanovic et al., 2007).

In addition to the impacts upon individual family members, most notably parents, there is undoubtedly family-level stress and change as the need arises to accommodate the afflicted young person's increased dependency. As a result, tensions may arise between members, and siblings may suffer reduced quality of life (Bowman et al., 2014) as parents need to invest more in the diagnosed relative.

Our group has been involved, for several decades, in the provision of services to families affected by the onset of mental health problems in a young relative. Our most well-developed and researched program of family support has been in early psychosis provided as one component of a comprehensive model of early intervention services for early psychosis through the Early Psychosis Prevention and Intervention Centre (EPPIC) at Orygen Youth Health in Melbourne, Australia (McGorry et al., 1996). From its inception EPPIC has focussed on the needs of families through the provision of structured psychoeducation programs and more intensive family therapy interventions for more complex cases. The model of care was differentiated from mainstream mental health care by focusing on early intervention, prevention of secondary trauma, and sensitivity to the early stages of illness (McGorry et al., 2010). The model has expanded over the preceding 5 years to include youth-friendly mental services for a broader range of disorders, most notably mood and anxiety disorders through the development of headspace centers (Rickwood et al., 2014). *Headspace* centers have been designed to provide accessible, youth-friendly, and evidence based “one-stop” services to address the mental health need of young people aged 12–25 years. Services include brief mental health care (maximum of 10 psychology sessions per year) to youth between the ages of 12 and 25 with high prevalence mental health problems (Rickwood et al., 2015). In keeping with the model of integration of clinical innovation and research at Orygen, our group developed and evaluated specific structured programs for carers. We have previously described our family intervention for first-episode psychosis carers as a key component of our EPISODE II study (Gleeson et al., 2010). The aim of the study was to reduce the onset of relapse in young people who had reached remission on positive symptoms of psychosis in the first year after commencement of treatment at EPPIC.

The manualised family intervention included a series of discrete modules which aimed to address the families' needs in terms of stress and psychoeducation in addition to targeting specific skills in problem solving and communication with their young relative.

Compared with usual specialist care at EPPIC, families who received the EPISODE II family intervention showed significant improvements in family stress levels, as conceptualized through the transactional model of stress and coping (Szmukler et al., 1996). In particular recipients of the intervention scored significantly higher in relation to positive appraisal of caregiving, suggesting that through the intervention they gained a greater appreciation of the difference they could make to the recovery of their young relative or a greater awareness of their strengths as carers. This was the first randomized controlled trial to demonstrate the effectiveness of an intervention specifically designed for FEP families.

Although the intervention has informed practice at EPPIC, sustaining and disseminating the manualized intervention has been challenging in line with the well-documented status quo of family interventions across the mental health service sector (Fadden, 1998; Lehman and Steinwachs, 1998; Eassom et al., 2014). The challenges to dissemination include procuring and maintaining the required resources for staff training and ongoing supervision. Stretching the precious resources of services to address the specific needs of families of young people who have reached the milestone of remission may also be a challenge when weighed against the needs of families facing the acute stage or those families challenged by persistent unremitting psychosis in their young relative. These challenges are similar of course for other mental health disorders that have their onset in youth. This means that important preventive opportunities may be lost.

In order to address this our team has turned to digital technology (Alvarez-Jimenez et al., 2016). With the initial aim of making evidence based interventions available for young people and carers within an online format, we perceived that digital technology provided affordances that extended beyond traditional face-to-face interventions (Alvarez-Jimenez and Gleeson, 2012). A process of 7 years of development and pilot evaluation has resulted in a suite of digital interventions for specific youth mental health populations, including two online carer applications. Specifically, we developed Meridian for carers of young people diagnosed with anxiety and depression and Altitudes for carers of young people diagnosed with early psychosis We describe our interventions here along with quantitative outcome data from a pilot study of Meridian for a group of carers with a relative diagnosed with depression. This pilot directly informed the development of Altitudes for carers of relatives recovering from early psychosis.

## Materials and methods

*Meridian and Altitudes* are both powered by our Moderated Online Social Therapy (MOST) software platform, which has provided a framework for six separate individual online platforms, including youth diagnosed with psychosis, at risk mental state, depression, help-seeking young people, and carers (Gleeson et al., 2012; Alvarez-Jimenez et al., 2013; Lederman et al., 2014).

MOST has evolved over the preceding 7 years to address specific challenges relating to the needs of people affected by youth mental health problems. The most significant challenge was to engage young people and carers over a period of many months so that the exposure to the intervention is matched with the necessary period of time for recovery, an outcome rarely achieved through online interventions. Second, problems of stigma are well-known factors in the avoidance of systems of care, which can also result in social isolation for carers and youth alike (Magliano et al., 2002). Thus, the intervention needed to foster social connectedness amongst carers of young people with mental-ill health. Finally, our carer interventions needed to extend beyond delivery of content because provision of information alone rarely changes behavior in families or improves their mental health outcomes (McFarlane et al., 2003; Lucksted et al., 2012).

Our approach to addressing these challenges has been underpinned by key conceptual frameworks. First, the principles of participatory design have required that we involve young people and carers in our team with regular consultation on their experience of using the systems (Wadley et al., 2013). We became aware of evidence highlighting that a focus on deficits in online systems can lead to demoralization and disengagement and so we began to specifically draw upon positive psychology frameworks, including concepts of family self-efficacy (Bandura et al., 2011; Alvarez-Jimenez et al., 2012). Third, we were strongly influenced by the supportive accountability framework, which was developed to operationalize the way in which human support enhances sustained engagement with online interventions. The supportive accountability theory has drawn together theoretical models, notably the self-determination theory of motivation, with empirical investigations of the role of human support in computer mediated interventions across various fields (Mohr et al., 2011). This highlighted the need to design computer mediated functions that equipped expert and peer moderators to interact with users in ways that matched with users' specific motivation for system involvement. We realized also that system functions needed to address fundamental needs in users to experience autonomy, competency, and connectedness, otherwise there was a high risk of disengagement and rapid drop-off in involvement (Ryan and Deci, 2000; Mohr et al., 2011). On the other hand, we saw specific opportunities in social media to facilitate connectedness among users who are often socially disconnected and stigmatized.

These frameworks required us to assemble an expanded team with additional capabilities and experience including in the fields of user experience, computer programing and web design, professional writers, graphic, and comic artists, in addition to our team of clinical psychologists and psychiatrists.

MOST evolved to integrate online therapy content with purpose built Facebook-style online social networking, and peer and expert moderation. Our carer applications, *Meridian (for depression)* and *Altitudes (for early psychosis)*, included specific content designed to addresses the needs of carers. These elements and functions were carefully integrated.

Our online therapy was provided through “steps and pathways.” For example, in Altitudes we included 10 separate pathways including content on self-care, psychoeducation, communication with the young person, and strategies to cope with stress related to caregiving. In line with the principle of autonomy, carers could work through the 39 small discrete “steps” in any order they wished. Each step was comprised of brief audio or text material integrated with graphics. Much of the content was initially derived from the EPISODE II study manual but there were significant innovations, most notably the addition of the character strengths approach from positive psychology in order to enhance competency in line with self-determination theory. Importantly, the user had the option of regularly engaging in commentary on the therapy material through a posting and comments function in order to create opportunities for personal reflection and to create an experience of undertaking the therapy within a supportive group environment. Each of the steps was also linked to specific actions that either facilitated skills practice (e.g., relaxation) or behavioral experiments (e.g., reappraisals of communication with one's relative). Actions could also be accessed independently of the therapy steps and were “recommended” to users in line with their identified personal strengths and needs.

The social networking functions enabled each user to set up a personalized profile similar to Facebook. Expert moderators, who were clinical psychologists with specific family experience in youth mental health settings, were identifiable as a separate class of users. Users could also post specific problems related to caring for their relative (or any issue) which, following clarification with a moderator, were addressed by groups of users through the “talk it out” function. This followed the discrete steps of problem solving therapy which has been utilized in multifamily therapy and behavioral family therapy (Falloon, 1988; McFarlane et al., 2003). Solutions were generated and remained available for review by all users facing similar challenges. Users could also set themselves discrete behavioral goals for a specific time frame (e.g., commit each day to self-care for the next month) and others could join them or support them in the “team up” challenge.

Communication and activity through the social networking was organized into a newsfeed which included posts to an individuals' walls, comments on therapy steps, and other activity that was initiated by users in the system.

Peer moderators, with lived experience of caring for a relative with severe mental health problems and several years of experience in providing face-to-face peer support at Orygen (including EPPIC) played a critical role in welcoming new users onto the system, modeling the use of the system online, providing emotional support, encouraging engagement, and by initiating online communication threads among users.

Expert moderators ascertained the specific motivations for family members to engage in the system, for example to learn more about depression (in Meridian) or psychosis (in Altitudes), or to be more connected with others facing shared challenges. At weekly supervision meetings online moderators presented formulations in line with self-determination theory with the aim of enhancing engagement and user self efficacy. Moderators carefully tracked patterns of engagement and recommended specific content through the system (e.g., specific steps) to individual users based on the assessment of their motivations to use the system. If engagement waned moderators could utilize an SMS system built into the platform to facilitate reconnection of participants.

Risk was managed at multiple levels (Gleeson et al., 2014). New users were carefully inducted into the system's terms of use including appropriate online communication. Inappropriate content was picked up through an automated screen, but users could also report inappropriate communication and moderators could delete posts if indicated. User accounts could be suspended if necessary and the team followed a detailed roster of checks and a risk assessment protocol.

### Meridian feasibility study

Meridian was evaluated in an initial feasibility study for carers with a young relative receiving treatment for depression or anxiety. We considered that this population of carers provided a useful basis for a feasibility trial that would inform our work with other carer populations, including early psychosis because: (a) they share similar demographic features including stage of family life; (b) they are faced with navigating access to mental health care for the first time; (c) they have had little previous exposure to psychoeducation or family support; and (d) they present with significant stress related to uncertainty regarding the day-to-day management of the young person's behavior.

Content specific to psychosis was not included but the study provided an important opportunity to assess feasibility and acceptability of the platform for carers. For the purposes of the study carers were recruited from a *headspace* center in the western suburbs of Melbourne.

The design comprised a single group study with a baseline assessment followed by a 3-month intervention phase, when carers had access 24 h per day to Meridian via any internet enabled device, and follow up assessments. The study was approved by the Human Research and Ethics Committee at The University of Melbourne.

Inclusion criteria for the study were: (a) a first degree relative, or legal guardian, of a current *headspace* consumer with a diagnosis of a mood or anxiety disorder in accordance with DSM-IV-TR (APA., 2000), or second degree relative residing with the consumer; (b) aged ≥ 16 years; (c) able to read and converse in English; and (d) a minimum of weekly contact with the identified consumer. Prospective participants were identified by *headspace* clinicians who requested permission from the carer to forward their contact details to the study research assistant. The study research assistant made contact in cases where this permission was provided to introduce the details of the study and to attain informed consent.

### Assessments

Assessments were conducted face-to-face and scheduled at baseline prior to induction into the application and at 3 months follow up. The primary outcome measure was acceptability and safety of the Meridian online program, assessed through a variety of measures and methods. Acceptability was defined as 50% of participants logging on at an average of more than once per week. The system was considered safe if there were no unlawful entries into the system, and was also indicated by lack of inappropriate use of the system by participants. Adverse incidents and all instances of inappropriate use of the system were also recorded. The back-end database recorded all instances of participant logins and page clicks, and the programming team monitored for unlawful entries.

Secondary outcomes were based upon a comparison of baseline and 3-month follow-up scores on measures of stress, depression, anxiety, psychological well-being and social support. The Perceived Stress Scale (PSS; Cohen and Williamson, 1988), a 10-item questionnaire that measures perceived stress over the preceding 3 months was used to measure stress. Satisfactory reliability estimates have been found in various populations on the 10-item PSS (Taylor, 2015). The 42-item version of Depression, Anxiety Stress Scale (DASS; 38) was used to measure anxiety and depression. All the DASS scales have demonstrated high internal consistency, convergent and discriminant validity and sensitivity to treatment effects (Brown et al., 1997). Psychological well-being was measured via the 42-item self-report questionnaire: Scales of Psychological Well-being (SPW; 39). The SPWB has demonstrated good internal consistency for the total score and each of the subscales (Ryff and Keyes, 1995). Confirmatory factor analysis has supported the theoretical six-factor model of well-being (Ryff and Keyes, 1995). Perceived Social support was measured by the Medical Outcomes Study: Social Support Survey (MOS-SSS) which is comprised of 19-items measuring perceived availability of functional social support (Sherbourne and Stewart, 1991). The MOS-SSS has shown strong psychometric properties including high internal consistency (0.91), test-retest reliability and sensitivity to change (Sherbourne and Stewart, 1991).

## Results

A total of 63 referrals were made to the study; 11 were ineligible, and 13 declined. A total of 30 participants were recruited and completed a baseline assessment and 29 completed baseline and at least a component of the follow up assessment. The nine remaining potential participants were unable to be contacted by the researcher before the end of the recruitment phase (i.e., did not respond to contact attempts by the Research Assistant before end of recruitment phase). Of the 29 relatives, 86% were female and there was a mean age at baseline of 47.76 (*SD* = 6.40) years. Twenty two participants (76%) were born in Australia. In terms of marital status, 13 were married (45%) 9 were divorced (31%), 3 were never married (10%) 1 was separated (3%), and 1 widowed (3%). Twenty eight participants (97%) were biological parents and one (3%) was a legal guardian.

In relation to the characteristics of the young people in their care 16 (55%) were female and 13 (45%) were male. The mean age of the young relatives was 16.83 (*SD* = 2.189) years. Diagnoses were reported by headspace clinicians in 26 of the 29 cases. These included 12 (48%) cases of major depressive disorder, 2 cases of generalized anxiety disorder (6%), 1 (3%) case of bipolar II disorder, 2 (6%) cases of obsessive compulsive disorder, 1 (3%) case of panic disorder, 2 (6%) cases of depression without a diagnoses being specified, 5 (17%) cases of depression combined with anxiety without a diagnosis being specified, 1 (3%) case of anxiety without a diagnosis being specified. According to the carers, all 29 young relatives had seen a psychologist at headspace in the preceding 3 months for treatment for depression or anxiety.

Importantly, 41% percent of the carers reported suffering from a chronic medical illness. These included diabetes, asthma, chronic fatigue, thyroid disorder, anxiety, and depression. In relation to education, 51% had not completed the final year of secondary school education and 17% listed a tertiary degree as their highest level of qualification.

### Feasibility, acceptability, and safety

In terms of primary outcomes, data were available on 27 participants at follow-up (93%). There were no adverse events associated with use of the system. There were no incidents of inappropriate posts by users onto the system identified by users or moderators or by the automated detection system.

From the 29 participants there was a total of 675 logins with a median of 11 per participant (range = 1–229). For each participant, we calculated their duration (in weeks) of involvement in Meridian by subtracting the end date (June 5, 2015) from the date of their baseline interview. The number of weeks of involvement ranged from 11 to 32 weeks. We then calculated the mean number of logins per week for each participant which was 0.9, just < 1 log on per week on average per participant. In terms of our a priori threshold, 30% logged on once or more per week on average, while 50% or more logged on once per fortnight. In terms of the breakdown of activity in the social networking functions, there was an average of 30 posts per participant, an average of 6 therapy “steps” undertaken and average of 5 therapy “actions” completed. A total of 22 online problem solving groups were completed.

### Secondary outcome variables

In relation to secondary outcomes (see Table 1), there was a statistically significant reduction on perceived stress on the PSS at follow-up [*t*_(26)_ = 3.23, *p* = 0.003, Cohen's *d* = 0.4]. In addition, the reliable change index (RCI) was calculated for each participant in accordance with Christensen and Mendoza (Christensen and Mendoza, 1986). The RCI is the standardized change score for each participant calculated by dividing the change score by the standard error of the change score. If the RCI is < 1.96 then the difference is not significant. In 18 (62%) cases the RCI was both positive and >1.96. In 3 (10%) of cases the RCI was negative and >1.96. As shown in Table 1, scores on other secondary outcomes measures were stable.

**Table 1:** **Means and standard deviations for baseline and follow-up measures**.

	**Time 1** ***n*** = **29**		**Time 2** ***n*** = **25**	
**Scale**	**M (*SD*)**	**Range**	**M (*SD*)**	**Range**
DASS total	36.19 (33.32)	0–126	35.71 (37.18)	0–132
DASS anxiety	9.28 (10.24)	0–42	8.65 (10.34)	0–33
DASS depression	12.31 (12.43)	0–42	13.31 (16.15)	0–64
PSS	21.86 (6.84)	11–35	19.07 (7.36)	8–33
MOS-SSS overall support index	3.83 (0.94)	1.94–5.00	4.00 (0.94)	1.83–5.00
SPW total	131.90 (37.75)	70–192	126.48 (37.83)	65–195

*DASS, Depression Anxiety Stress Scale; PSS, Perceived Stress Scale; MOs-SSS, Medical Outcomes Study: Social Support Survey; SPW Total, Scales of Psychological Well-being Total*.

### Associations between system usage and outcome variables

Interestingly there were moderate correlations with reductions in stress and use of the system. Specifically, there were moderate and significant correlations between degree of improvement in stress and number of log-ons (*r* = 0.548, *p* = 0.003) and number of pathways commenced (*r* = 0.453, *p* = 0.02).

## Discussion

Researchers over many decades have been successful in developing and evaluating interventions for carers with relatives afflicted by mental health disorders, including psychosis. Our group contributed to the evidence-base of effective interventions suitable for FEP carers through our EPISODE II study, but we are mindful of the long history of challenges in translating evidence-based interventions into accessible and sustainable treatment options (Lehman, 2000)—challenges which are important in other youth mental health disorders such as depression because families are in the throes of adapting to the first onset.

Our MOST application for carers provides one approach to solving this problem. Our applications include interventions tailored for carers of youth affected by early psychosis and depression and anxiety that integrate tailored therapy content, expert and peer moderation, and social networking to create an online therapeutic environment that can be accessed by carers across wide geographical regions, including remote and regional locations. In addition, it can be utilized by carers at a time and location convenient to them wherever an Internet enabled device is available.

The *Meridian* feasibility study indicated that the MOST system was safe for carers of young people facing mental health problems. Although our a prior threshold of 50% or more of participants logging on once per week was not quite attained, we noted that a high proportion of participants were motivated to maintain regular contact with the system, albeit at a lower frequency than we predicted. This may simply reflect carers' preference for use and their busy lives. The severity of illness was moderate to severe in line with the *headspace* model, which may have affected the perceived need of carers to engage in the system.

In addition, we have found promising reductions in family stress and correlations between those reductions and use of our system. In terms of patterns of use, we note that carers have taken to using both social networking features in addition to online therapy content, which is encouraging in terms of the design of our system, and provides support for our approach to participatory design and is in keeping with the supportive accountability model.

However, the design of the study did not enable conclusions to be drawn regarding the effectiveness of the intervention for carers. Given the results we have been encouraged to consider the potential for the application for carers of young people recovering from early psychosis. Our experience with *Meridian* also informed modifications, including: (1) a more considered approach to the face-to-face induction of users into the system, and (2) more careful assessment of the specific motivations of each user to use the system to optimize engagement.

To address the question of whether Altitudes is effective is reducing levels of stress in carers of young people diagnosed with psychosis we have initiated a cluster RCT comparing stress outcomes in carers randomized to usual care at EPPIC plus the Altitudes application, vs. usual care alone, with research assistants undertaking assessments kept blind to treatment allocation (Gleeson et al., 2017). Families comprise the cluster. In order to address a broader scope of carer need in FEP we have widened the inclusion criteria that we utilized in the EPISODE II trial to include all carers regardless of the remission status of their relative. Eligible participants include carers (namely parent, grandparent, siblings, and partners) of young people (aged 15–27 years inclusive) who: (1) are currently receiving treatment for FEP at EPPIC; or, (2) have recently been discharged by their treating team after an episode of care at EPPIC. There is no specified limit on the number of eligible participants from each family.

The primary outcome in the Altitudes trial is carers' perceived stress as measured by the PSS (Cohen and Williamson, 1988) at 3 and 6 months after treatment allocation.

In order to gain a more dynamic picture of carer stress, participants utilize our Smartphone Ecological Momentary Assessment (SEMA) tool. The SEMA tool is readily downloadable to participants running Android or iOS operating systems on their smartphone. The tool delivers surveys (administered following the baseline and 6 month assessment time point) at eight time points per day in the waking hours of each participant for a period of 1 week. The survey questions include items regarding interactions between the carer and their relative suffering FEP since the preceding survey, perceived behavioral problems in their relative, attributions for perceived behavioral problems, coping strategies (Cotton et al., 2013), perceived parental self-efficacy, stress and depression, and perceived social support. Participants are prompted to complete SEMA surveys every 90 min (±30 min) over a 12 h period (e.g., 9 a.m. to 9 p.m.) each day for 7 consecutive days.

As of 21st December, 2016, 100 participants have been randomized into the trial with the aim of recruiting 160.

Our cluster randomized trial, currently in progress, has been designed to address the important question of whether our intervention is effective in reducing stress related to caregiving for FEP family members. If so, our MOST framework will address a critical problem that has beleaguered the field over the preceding decades—how to sustain the availability of evidence-based care for family members at a critical phase in early psychosis. Our ultimate hope is that this will prevent long-term stress and health impacts upon family members providing care in highly difficult circumstances.

## Ethics statement

This study was carried out in accordance with the recommendations of The University of Melbourne Human Research and Ethics Committee with written informed consent from all subjects. This study was carried out in accordance with the National Statement on Ethical Conduct in Research Involving Humans (2007), published by the National Health and Medical Research Council of Australia. All subjects gave written informed consent in accordance with the Declaration of Helsinki. The protocol was approved by the Human Research and Ethics Committee of The University of Melbourne.

## Author contributions

JG led the development of the design, analysis and led the drafting of the manuscript. MA co-led the development of the design. RL, PK, GW, SB, SC, HH, KC, and MA made significant contributions to the design of the work. All authors were involved in the drafting or revising of the manuscript, all authors approved the final version and are agreeable to be accountable for the work.

## Funding

This research was funded by a Service Improvement Project Grant administered by headspace National Office and by The Australian Government's Collaborative Research Networks (CRN) program. The administer of the funds is The Australian Catholic University. The CRN had no involvement in the design of the study or the collection of data. SC is supported by a NHMRC Career Development Fellowship (APP1061998). MA is supported by a NHMRC Career Development Fellowship (APP1082934).

### Conflict of interest statement

The authors declare that the research was conducted in the absence of any commercial or financial relationships that could be construed as a potential conflict of interest. The reviewer JS and the handling Editor declared their shared affiliation, and the handling Editor states that the process nevertheless met the standards of a fair and objective review.

## References

[B1] APA (2000). Diagnostic and Statistical Manual Of Mental Disorders, 4th TR Edn. Washington, DC: American Psychiatric Association.

[B2] Addington J. Coldham E. L. Jones B. Ko T. Addington D. (2003). The first episode of psychosis: the experience of relatives. Acta Psychiatr. Scand. 108, 285–289. 10.1034/j.1600-0447.2003.00153.x 12956829

[B3] Alvarez-Jimenez M. Bendall S. Lederman R. Wadley G. Chinnery G. Vargas S.. (2013). On the HORYZON: moderated online social therapy for long-term recovery in first episode psychosis. Schizophr. Res. 143, 143–149. 10.1016/j.schres.2012.10.009 23146146

[B4] Alvarez-Jimenez M. Gleeson J. F. (2012). Connecting the dots: twenty-first century technologies to tackle twenty-first century challenges in early intervention. Aust. N. Z. J. Psychiatry 46, 1194–1196. 10.1177/0004867412464067 23212140

[B5] Alvarez-Jimenez M. Gleeson J. F. Bendall S. Lederman R. Wadley G. Killackey E.. (2012). Internet-based interventions for psychosis: a sneak-peek into the future. Psychiatr. Clin. North Am. 35, 735–747. 10.1016/j.psc.2012.06.011 22929876

[B6] Alvarez-Jimenez M. Gleeson J. F. Rice S. Gonzalez-Blanch C. Bendall S. (2016). Online peer-to-peer support in youth mental health: seizing the opportunity. Epidemiol. Psychiatr. Sci. 25, 123–126. 10.1017/S2045796015001092 26880239 PMC6998680

[B7] Bandura A. Caprara G. V. Barbaranelli C. Regalia C. Scabini E. (2011). Impact of family efficacy beliefs on quality of family functioning and satisfaction with family life. Appl. Psychol. 60, 421–448. 10.1111/j.1464-0597.2010.00442.x

[B8] Bowman S. Alvarez-Jimenez M. Wade D. Howie L. McGorry P. (2014). The impact of first episode psychosis on sibling quality of life. Soc. Psychiatry Psychiatr. Epidemiol. 49, 1071–1081. 10.1007/s00127-013-0817-5 24448630

[B9] Brown T. A. Chorpita B. F. Korotitsch W. Barlow D. H. (1997). Psychometric properties of the Depression Anxiety Stress Scales (DASS) in clinical samples. Behav. Res. Ther. 35, 79–89. 10.1016/S0005-7967(96)00068-X 9009048

[B10] Christensen L. Mendoza J. L. (1986). A method of assessing change in a single subject - an alteration of the rc index. Behav. Ther. 17, 305–308. 10.1016/S0005-7894(86)80060-0

[B11] Cohen S. Williamson G. M. (1988). Perceived stress in a probability sample of the United-States, in Social Psychology of Health, eds Shirlynn S. Stuart O. (Thousand Oaks, CA: Sage Publications, Inc), 31–67.

[B12] Cotton S. M. McCann T. V. Gleeson J. F. Crisp K. Murphy B. P. Lubman D. I. (2013). Coping strategies in carers of young people with a first episode of psychosis. Schizophr. Res. 146, 118–124. 10.1016/j.schres.2013.02.008 23490761

[B13] Damjanovic A. K. Yang Y. Glaser R. Kiecolt-Glaser J. K. Nguyen H. Laskowski B.. (2007). Accelerated telomere erosion is associated with a declining immune function of caregivers of Alzheimer's disease patients. J. Immunol. 179, 4249–4254. 10.4049/jimmunol.179.6.4249 17785865 PMC2262924

[B14] de Girolamo G. Dagani J. Purcell R. Cocchi A. McGorry P. D. (2012). Age of onset of mental disorders and use of mental health services: needs, opportunities and obstacles. Epidemiol. Psychiatr. Sci. 21, 47–57. 10.1017/S2045796011000746 22670412

[B15] Eassom E. Giacco D. Dirik A. Priebe S. (2014). Implementing family involvement in the treatment of patients with psychosis: a systematic review of facilitating and hindering factors. BMJ Open 4, 10. 10.1136/bmjopen-2014-006108 25280809 PMC4187461

[B16] Fadden G. (1998). Family intervention in psychosis. J. Mental Health 7, 115–122. 10.1080/09638239818166

[B17] Falloon I. R. (1988). Handbook of Behavioral Family Therapy. New York, NY: Guilford Press.

[B18] Gleeson J. F. Jackson H. J. Stavely H. Burnett P. (1999). Family intervention in early psychosis, in The Recognition and Management of Early Psychosis: A Preventive Approach, eds McGorry P. D. Jackson H. J. (New York, NY: Cambridge University Pres), 376–406.

[B19] Gleeson J. F. Lederman R. Wadley G. Bendall S. McGorry P. D. Alvarez-Jimenez M. (2014). Safety and privacy outcomes from a moderated online social therapy for young people with first-episode psychosis. Psychiatr. Serv. 65, 546–550. 10.1176/appi.ps.201300078 24687106

[B20] Gleeson J. F. M. Alvarez-Jimenez M. Lederman R. (2012). Moderated online social therapy for recovery from early psychosis. Psychiatr. Serv. 63:719. 10.1176/appi.ps.20120p719 22752039

[B21] Gleeson J. F. M. Cotton S. M. Alvarez-Jimenez M. Wade D. Crisp K. Newman B.. (2010). Family outcomes from a randomized control trial of relapse prevention therapy in first-episode psychosis. J. Clin. Psychiatry 71, 475–483. 10.4088/JCP.08m04672yel 20021994

[B22] Gleeson J. Lederman R. Herrman H. Koval P. Eleftheriadis D. Bendall S.. (2017). Moderated online social therapy for carers of young people recovering from first-episode psychosis: study protocol for a randomised controlled trial. Trials 18:27. 10.1186/s13063-016-1775-5 28095883 PMC5240433

[B23] Jansen J. E. Gleeson J. Cotton S. (2015). Towards a better understanding of caregiver distress in early psychosis: a systematic review of the psychological factors involved. Clin. Psychol. Rev. 35, 59–66. 10.1016/j.cpr.2014.12.002 25531423

[B24] Lederman R. Wadley G. Gleeson J. Bendall S. Alvarez-Jimenez M. (2014). Moderated online social therapy: designing and evaluating technology for mental health. ACM Trans. Comput. Hum. Interact. 21, 1–26. 10.1145/2513179

[B25] Lehman A. F. (2000). Commentary: what happens to psychosocial treatments on the way to the clinic? Schizophr. Bull. 26, 137–139. 10.1093/oxfordjournals.schbul.a033431 10755674

[B26] Lehman A. F. Steinwachs D. M. (1998). Patterns of usual care for schizophrenia: initial results from the Schizophrenia Patient Outcomes Research Team (PORT) client survey. Schizophr. Bull. 24, 11–32. 10.1093/oxfordjournals.schbul.a033303 9502543

[B27] Lucksted A. McFarlane W. Downing D. Dixon L. Adams C. (2012). Recent developments in family psychoeducation as an evidence-based practice. J. Marital Fam. Ther. 38, 101–121. 10.1111/j.1752-0606.2011.00256.x 22283383

[B28] Magliano L. Marasco C. Fiorillo A. Malangone C. Guarneri M. Maj M.. (2002). The impact of professional and social network support on the burden of families of patients with schizophrenia in Italy. Acta Psychiatr. Scand. 106, 291–298. 10.1034/j.1600-0447.2002.02223.x 12225496

[B29] Marshall M. Lewis S. Lockwood A. Drake R. Jones P. Croudace T. (2005). Association between duration of untreated psychosis and outcome in cohorts of first-episode patients: a systematic review. Arch. Gen. Psychiatry 62, 975–983. 10.1001/archpsyc.62.9.975 16143729

[B30] McFarlane W. R. Dixon L. Lukens E. Lucksted A. (2003). Family psychoeducation and schizophrenia: a review of the literature. J. Mar. Fam. Ther. 29, 223–245. 10.1111/j.1752-0606.2003.tb01202.x 12728780

[B31] McGorry P. D. Edwards J. Mihalopoulos C. Harrigan S. M. Jackson H. J. (1996). EPPIC: an evolving system of early detection and optimal management. Schizophr. Bull. 22, 305–326. 10.1093/schbul/22.2.305 8782288

[B32] McGorry P. D. Nelson B. Goldstone S. Yung A. R. (2010). Clinical staging: a heuristic and practical strategy for new research and better health and social outcomes for psychotic and related mood disorders. Can. J. Psychiatry Rev. Can. Psychiatrie 55, 486–497. 10.1177/070674371005500803 20723276

[B33] Mohr D. C. Cuijpers P. Lehman K. (2011). Supportive accountability: a model for providing human support to enhance adherence to ehealth interventions. J. Med. Internet Res. 13:e30. 10.2196/jmir.1602 21393123 PMC3221353

[B34] Onwumere J. Bebbington P. Kuipers E. (2011). Family interventions in early psychosis: specificity and effectiveness. Epidemiol. Psychiatr. Sci. 20, 113–119. 10.1017/S2045796011000187 21714356

[B35] Patterson P. Birchwood M. Cochrane R. (2005). Expressed emotion as an adaptation to loss: prospective study in first-episode psychosis. Br. J. Psychiatry Suppl. 48, s59–s64. 10.1192/bjp.187.48.s59 16055810

[B36] Rickwood D. J. Telford R. R. Mazzer K. R. Parker A. G. Tanti C. J. McGorry P. D. (2015). The services provided to young people by headspace centres in Australia. Med. J. Aust. 202, 533–536. 10.5694/mja14.01695 26021365

[B37] Rickwood D. Telford N. McGorry P. Hetrick S. Killackey E. (2014). Evaluation innovation for early psychosis headspace services. Early Interv. Psychiatry. 8, 127

[B38] Ryan R. M. Deci E. L. (2000). Self-determination theory and the facilitation of intrinsic motivation, social development, and well-being. Am. Psychol. 55, 68–78. 10.1037/0003-066X.55.1.68 11392867

[B39] Ryff C. D. Keyes C. L. M. (1995). The structure of psychological well-being revisited. J. Pers. Soc. Psychol. 69, 719–727. 10.1037/0022-3514.69.4.719 7473027

[B40] Sherbourne C. D. Stewart A. L. (1991). The MOS Social Support Survey. Soc. Sci. Med. 32, 705–714. 10.1016/0277-9536(91)90150-B 2035047

[B41] Sobieraj M. Williams J. Marley J. Ryan P. (1998). The impact of depression on the physical health of family members. Br. J. Gen. Practice 48, 1653–1655. 10071397 PMC1313239

[B42] Szmukler G. I. Burgess P. Herrman H. Benson A. (1996). Caring for relatives with serious mental illness: the development of experience of caregiving inventory. Soc. Psychiatry Psychiatr. Epidemiol. 31, 137–148. 10.1007/BF00785760 8766459

[B43] Taylor J. M. (2015). Psychometric analysis of the ten-item perceived stress scale. Psychol. Assess. 27, 90–101. 10.1037/a0038100 25346996

[B44] Wadley G. Lederman R. Gleeson J. Alvarez-Jimenez M. (eds.). (2013). Participatory design of an online therapy for youth mental health, in Proceedings of the 25th Australian Computer-Human Interaction Conference: Augmentation, Application, Innovation, Collaboration, OzCHI, 2013.

